# Evaluation of a novel secondary check tool for intensity‐modulated radiotherapy treatment planning

**DOI:** 10.1120/jacmp.v15i5.4990

**Published:** 2014-09-08

**Authors:** Jonas D. Fontenot

**Affiliations:** ^1^ Department of Physics Mary Bird Perkins Cancer Center Baton Rouge LA USA

**Keywords:** intensity‐modulated radiation therapy, volumetric‐modulated arc therapy, treatment planning systems, quality assurance

## Abstract

The purpose of this study was to assess the accuracy and efficacy of an automated treatment plan verification, or “secondary check”, tool (Mobius3D), which uses a reference dataset to perform an independent three‐dimensional dose verification of the treatment planning system (TPS) dose calculation and assesses plan quality by comparing dose‐volume histograms to reference benchmarks. The accuracy of the Mobius3D (M3D) system was evaluated by comparing dose calculations from IMRT and VMAT plans with measurements in phantom geometries and with TPS calculated dose distributions in prostate, lung, and head and neck patients (ten each). For the patient cases, instances of DVH limits exceeding reference values were also recorded. M3D showed agreement with measured point and planar doses that was comparable to the TPS in phantom geometries. No statistically significant differences (p<0.05) were noted. M3D dose distributions from VMAT plans in patient cases were in good agreement with the TPS, with an average of 99.5% of dose points showing $\gamma_{5\%,3\text{mm}}<1$. The M3D system also identified several plans that had exceeded dose‐volume limits specified by RTOG protocols for those sites. The M3D system showed dosimetric accuracy comparable with the TPS, and identified several plans that exceeded dosimetric benchmarks. The M3D system possesses the potential to enhance the current treatment plan verification paradigm and improve safety in the clinical treatment planning and review process.

PACS number: 87.55.D‐, 87.55.km, 87.55.Qr,

## I. INTRODUCTION

With conventional (e.g., unmodulated) treatment fields, the quality of each treatment planning system (TPS) calculation was verified through the use of independent treatment plan verification (also referred to as “secondary check”) systems that typically apply simplistic scatter and pathlength corrections to calculations of dose to a single point for the purpose of independently verifying TPS monitor units.^(1)^ However, the complexity of dose distributions has increased substantially since the initial adoption of such methods. Inverse planning and intensity modulation of treatment fields have enabled the clinician to create highly conformal and irregular dose distributions that improve normal tissue sparing.^2^, ^3^ Subsequent developments in treatment planning and delivery that have improved efficiency, such as volumetric‐modulated arc therapy,^(4)^ have further reduced the technical obstacles of utilization and resulted in marked proliferation of these technologies over the last 10 to 15 years.^5^, ^6^ In most clinics in the United States, the current approach to dosimetric verification of such plans is the combination of a “secondary check” calculation to independently evaluate the accuracy of the TPS dose calculation algorithm, and a patient‐specific quality assurance (QA) measurement in a dedicated phantom to ensure data transfer fidelity and deliverability of the treatment plan. As the present work applies only to “secondary check” calculations, patient‐specific QA measurements and approaches will not be discussed further.

Despite increased complexity of treatment planning dose distributions, the methods and algorithms employed by “secondary check” systems have remained largely unchanged: dose calculations to a single point using simple heterogeneity corrections. Such limitations inevitably lower the ability of those systems to detect clinically meaningful errors in the TPS calculation throughout the high‐dose volume. An additional limitation of the current “secondary check” paradigm of calculating dose to a single point, aside from precluding a full three‐dimensional verification of the TPS accuracy, also precludes an independent assessment of the quality of the treatment plans with respect to dosimetric benchmarks. As a result, there is a potential role for more sophisticated treatment planning dose verification tools to enhance current clinical practice.

Recently, a new commercial secondary check tool has been developed (Mobius3D; Mobius Medical Systems, LP, Houston, TX). The system is intended to enhance the current secondary check paradigm by 1) performing an independent 3D calculation of the treatment plan within the patient CT geometry that allows for more comprehensive evaluation of TPS accuracy and its impact on the planning goals, and 2) evaluating of the quality of each treatment plan with respect to established dosimetric benchmarks. The potential benefit of such a system is to improve the value of secondary check calculations in validating TPS accuracy.

Despite the potential of the system, clinical validation of the system was not heretofore reported. The purpose of this work was to evaluate the dosimetric accuracy and efficacy of the system for clinical use. The accuracy of the Mobius3D (M3D) system was evaluated by comparing its dose calculations of IMRT and/or VMAT plans with 1) measured doses in phantom geometries, and 2) dose calculations from a commercial TPS in actual patient data. Efficacy was evaluated by assessing the ability of the system to automatically review treatment plan quality and identify instances of dosimetric deviations beyond configured tolerances.

## II. MATERIALS AND METHODS

### A. Overview of Mobius3D

M3D uses a collapsed cone convolution/superposition algorithm developed by the manufacturer to calculate dose in the patient or phantom geometry. Similar to convolution/superposition algorithms used by other commercial treatment planning systems, the M3D algorithm models the essential elements of the linear accelerator treatment head (e.g., MLC, jaws, flattening filter) and calculates the dose at each point within the patient by convolving the energy fluence with a dose deposition kernel. Because the algorithm is implemented on a graphical processing unit (GPU), calculations are purported to require less time compared to the existing TPSs that utilize a similar dose calculation algorithm.^(7)^

M3D operates on DICOM objects (CT images, RT Dose, RT Structure, and RT Plan) exported from the TPS following the completion of a patient treatment plan. Upon receiving the necessary files, the system associates the various objects under a patient‐specific entry and extracts the necessary treatment field information from the RT plan file. The treatment field information is then passed to the dose calculation algorithm, which uses this information to calculate the three‐dimensional dose distribution within the CT dataset associated with the plan. The dose calculation algorithm arrives precommissioned with a standard reference dataset specific to each linear accelerator manufacturer and model. The user has the ability to customize the model using a subset of site‐specific depth‐dose values and off‐axis ratios, though the stock reference model was utilized for this work. Following dose calculation, the dose distribution calculated by M3D is compared with the TPS dose extracted from the RT Dose file using dose‐volume histograms (DVH) of the structures associated with the CT dataset, isodose overlays on the CT dataset, and a 3D comparison of the dose matrices using the gamma metric.^(8)^ Both calculated sets of DVH profiles are automatically checked against reference DVH limits by using regular expressions of regions‐of‐interest (ROI) names to identify relevant structures within the plan and looking up available RTOG protocol dose limits for those structures. The user may also edit, remove, or add additional DVH limits.

### B. Phantom plans

The accuracy of the M3D system was evaluated in a phantom by comparing its dose calculations for IMRT and VMAT plans with measurements previously reported by our group.^(9)^ The four structure sets provided by Task Group 119 of the American Association of Physicists in Medicine^(10)^ (prostate, C‐shape, multitarget, and head and neck) were copied to a cylindrical solid water phantom, and IMRT and VMAT plans were constructed to meet the dosimetric goals specified in TG‐119 using a commercial treatment planning system (Pinnacle^3^, Philips Medical Systems, Fitchburg, WI). Additional details regarding the treatment planning parameters and results are described elsewhere.^(9)^ Point‐dose measurements were performed in high‐ and low‐dose regions using an A1SL cylindrical type ionization chamber. Planar dose measurements were performed in the sagittal and coronal planes of the phantom using radiochromic film. Additional details of the experimental geometry and dosimetry techniques are also described elsewhere.^(9)^

The treatment planning data (CT images, RT Dose, RT Structure, and RT Plan files) from the TG‐119 plans was exported to the M3D server (running version 1.2.1 of the software), which then performed its own calculation of the three‐dimensional dose distribution within the phantom resulting from the planned treatment fields. The sagittal and coronal planar doses corresponding to the film plane of each plan were extracted from the M3D and TPS dose distributions and registered to the planar dose measured with film using in‐house code (MATLAB, MathWorks, Natick, MA). The gamma metric^(8)^ was used to quantify the agreement between the calculated (M3D and TPS) and measured planar doses using criteria of 3% dose difference and 3 mm distance to agreement. M3D and TPS point doses were compared with measured values by taking the mean dose of a region of interest (ROI) encompassing the volume of the chamber at the location of the measurement. Percentage differences between point doses were computed using the formula recommended in AAPM Task Group 119. Differences in agreement between the M3D and TPS calculations with measured planar and point doses was assessed for significance (p<0.05) using the Wilcoxon signed‐rank test.

### C. Patient plans

The accuracy of the M3D system was also evaluated by comparing its dose calculations from VMAT plans in actual patient data with that calculated by the TPS. The study utilized clinical VMAT treatment planning data for ten patients with prostate cancer, ten patients with lung cancer, and ten patients with head and neck cancer, which included simultaneous irradiation of regional lymphatics. All VMAT plans consisted of one (prostate patients) or two arcs (all others), utilized an energy of 6 M V, a collimator angle of 45°, a leaf motion constraint of 2 mm (lung patients) or 4 mm (all others) per degree of gantry rotation, and were constructed for delivery on an Elekta linear accelerator (Infinity; Elekta AB, Stockholm, Sweden). The plans were exported to the M3D server, which performed its own calculation of the three‐dimensional dose distribution within the patient CT data resulting from the planned treatment fields. As one of its evaluation metrics, M3D calculates the percentage of points showing gamma values less than one using default criteria of 5% dose difference and 3 mm distance to agreement. The 5% dose threshold follows the recommendation of AAPM Task Group 40^(11)^ for the agreement between primary and verification calculations when using sophisticated algorithms, substantial field blocking, or heterogeneity corrections. This choice is further supported by the recent AAPM Task group 114 report,^(1)^ which recommended a 3% tolerance between similar algorithms used to calculate dose in the patient geometry for non‐IMRT fields. The average percentage of points showing gamma less than one was computed for each site and over all sites. Instances of DVH values exceeding reference values were also recorded.

## III. RESULTS

### A. Phantom plans

Percentage differences between the calculated (M3D and TPS) and measured point doses for the TG‐119 structure sets are shown in Table 1. In general, the TPS and M3D showed similar agreement with point doses measured with an ionization chamber. For IMRT point dose comparisons, Mobius3D showed slightly better agreement compared with the measurement (‐0.6% vs. ‐0.8%,p=1); however, taking the average agreement irrespective of the sign of the differences showed slightly better agreement with measurement in favor of the TPS (1.5% vs. 2.2%, p=0.13). However, neither of these differences was found to be statistically significant. Despite similar means, Mobius3D showed a larger standard error in point‐dose differences; all TPS doses were within 3% of the measured dose, whereas M3D point doses showed differences of between 3% and 5% in four cases. For VMAT point‐dose comparisons, Mobius3D again showed slightly better average agreement with measurement (−1.6 vs. −1.9,p=0.71), with slightly better agreement in favor of the TPS when neglecting the sign difference (2.0% vs. 2.3%, p=0.28). Again, neither of these differences was found to be statistically significant. For VMAT plans, the TPS and Mobius3D showed similar standard errors in point‐dose differences.

Results of the comparison between the planar dose calculations and the film measurements are shown in Table 2. In general, the TPS and M3D showed similar agreement with planar doses measured with radiochromic film. For IMRT plans, the TPS showed slightly better average agreement with measurement (96.9% vs. 96.2%). Conversely, M3D showed slightly better average agreement with measurement (97.5% vs. 97.0%) for VMAT plans. However, neither of these differences was found to be statistically significant (p=0.20 and 0.06 for IMRT and VMAT, respectively). The standard error of the agreement with film measurements was similar for both the TPS and M3D for VMAT plans. For both dose calculations and delivery types, agreement with film was highest for the mock prostate plans (range: 99.7%–100%) and lowest for the mock head and neck plans (range: 90.9%–95.6%).

**Table 1:** Measured point doses and percent differences between doses measured and calculated by the treatment planning system (TPS) and Mobius3D (M3D) for IMRT and VMAT treatment plans of the AAPM Task Group 119 structure sets. Percent differences are displayed as the mean ±standard error (N=5)

	*IMRT*			*VMAT*		
		*% diff*			*% diff*	
*Location*	*Measured Dose (cGy)*	*TPS*	*M3D*	*Measured Dose (cGy)*	*TPS*	*M3D*
*Multitarget*						
Central target	213.9±0.3	‐0.4±0.1	‐0.6±0.1	219±0.2	0.3±0.1	1.5±0.1
Superior target	118.5±0.8	‐0.6±0.4	‐3.3±0.4	108±0.2	‐0.1±0.1	‐0.5±0.1
Inferior target	59.8±0.5	‐2.8±0.2	‐0.1±0.2	53.7±0.2	‐1.2±0.1	‐1.2±0.1
*Prostate*						
PTV	182.6±0.2	‐0.8±0.1	‐0.2±0.1	184.3±0.3	‐0.6±0.2	0.2±0.2
Rectum	134.4±0.5	‐1.6±0.3	2.5±0.3	144.0±0.3	‐1.5±0.2	1.7±0.2
Bladder	138.8±0.8	1.3±0.4	‐1.2±0.4	129.4±0.8	‐2.8±0.4	‐4.2±0.4
*Head and neck*						
PTV	207.1±0.1	‐2.9±0.0	‐3.5±0.0	198.0±0.3	‐4.2±0.1	‐3.5±0.1
Spinal cord	124.1±1.4	‐1.4±0.7	‐2.0±0.7	127.4±0.9	‐4.0±0.4	‐2.8±0.4
*C‐shape*						
Central core	53.2±0.3	‐0.9±0.2	‐3.4±0.2	44.0±0.3	‐2.0±0.1	‐4.0±0.1
Outer target	212.0±0.3	1.8±0.1	5.5±0.1	202.0±0.7	‐2.8±0.3	‐3.5±0.3
Average		‐0.8±0.5	‐0.6±0.9		‐1.9±0.5	‐1.6±0.7
|Average|		1.5±0.3	2.2±0.6		2.0±0.5	2.3±0.5

**Table 2:** The percentage of calculated treatment planning system (TPS) and Mobius3D (M3D) dose points with gamma (γ) values less than one when compared with dose points measured with radiochromic film for IMRT and VMAT plans of the AAPM Task Group 119 structure sets. Percentage of points are shown as the mean ±standard error (N=3)

	$\%\;\text{points}\;\gamma_{3\%,3\text{mm}}<1$			
	*IMRT*		*VMAT*	
*Film Plane*	*TPS*	*M3D*	*TPS*	*M3D*
*Multitarget*				
Coronal	98.6±0.3	96.0±0.6	97.4±0.2	99.0±0.2
Sagittal	98.6±0.6	96.4±0.7	98.1±0.3	98.5±0.3
*Prostate*				
Coronal	99.7±0.2	99.8±0.1	100±0.0	99.9±0.0
Sagittal	99.5±0.2	99.9±0.1	99.9±0.1	100±0.0
*Head and neck*				
Coronal	95.6±0.4	94.3±0.4	90.9±0.2	91.8±0.2
Sagittal	91.6±0.2	91.4±0.3	94.2±0.3	94.8±0.1
*C‐shape*				
Coronal	95.5±0.8	96.7±0.8	98.7±0.1	98.4±0.3
Sagittal	96.3±0.8	94.8±0.6	96.8±0.1	97.4±0.6
Average	96.9±1.0	96.2±1.0	97.0±1.1	97.5±1.1

### B. Patient plans

**Figure 1:**
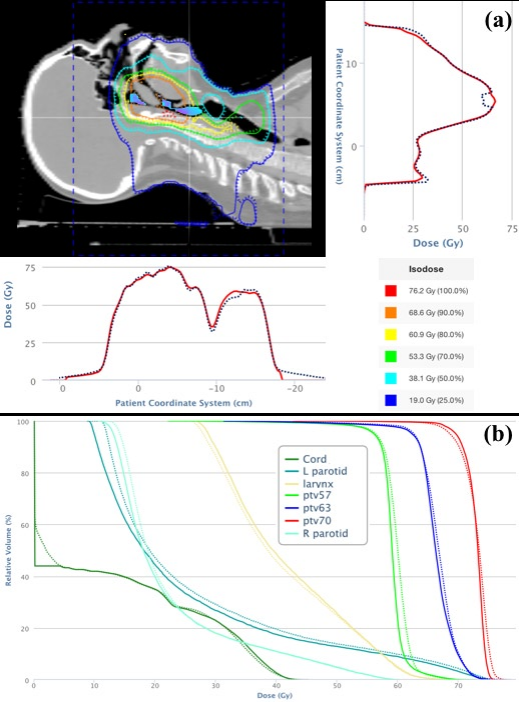
Isodose distributions and dose profiles (a) from the treatment planning system (solid lines) and Mobius3D (dashed lines) for head and neck Case #6. Regions of blue and purple colorwash in the sinus and airway denote regions where M3D underpredicted the TPS dose by greater than the gamma criteria. Dose‐volume histograms (b) for relevant regions of interest calculated by the TPS (solid) and M3D (dashed). (Adapted from the M3D user interface.)

The percentage of M3D‐calculated dose points in patient cases showing a gamma values less than one (i.e., γ5%,3mm<1) when compared with the TPS is shown in Table 3. A representative result is shown in Fig. 1 for a head and neck patient. On average, 100% (range: 99.9%–100%), 99.7% (range: 99.0%–100%), and 98.7% (range: 93.2%–99.9%) of M3D dose points showed gamma values less than one for prostate, lung, and head and neck plans, respectively. For the lung and head and neck cases, the anatomical region most typically associated with regions of gamma failure was the trachea and esophagus, each of which contained significant volumes of air (see Fig. 1). In these volumes, M3D calculated a slightly (approximately 5%–7%) lower in dose to air compared with surrounding tissue, as compared with the TPS. Theoretical considerations and previous publications^12^, ^13^ suggest that absorbed dose should be lower in air than surrounding tissue; however, it is likely that the TPS interpolates the dose in this region due to the comparative lack of clinical relevance of the absorbed dose to air. Doses calculated by M3D in low‐density lung tissue were found to be within tolerance criteria of the TPS calculation.

M3D also reported several instances of TPS‐calculated or M3D‐calculated (or both) DVHs exceeding the default dosimetric benchmarks. For the prostate patients, dose volumes that had been potentially exceeded were identified for the femoral heads (limits specified in RTOG 0822), bladder (RTOG 0126), and penile bulb (RTOG 0126). For the lung cases, dose volumes that had been potentially exceeded were identified for the spinal cord (RTOG 0623) and its planning organ‐at‐risk volume (PRV) and the esophagus (RTOG 0920). Finally, for the head and neck patients, dose volumes that had been potentially exceeded were identified for the one or both parotid glands (RTOG 0912) and the mandible (RTOG 0225). In all cases, the final dose distributions and dose volumes had previously been thoroughly reviewed and approved as clinically acceptable by the radiation oncologist; nevertheless, these warnings provided a useful tool for ensuring that dose‐volume limits had previously been reviewed and approved during the clinical treatment planning process.

**Table 3:** The percentage of dose points calculated by the treatment planning system (TPS) with gamma (γ) values less than one when compared with those calculated by Mobius3D (M3D) for VMAT plans for cancers of the prostate, lung, and head and neck

	$\%\;\text{points}\;\gamma_{5\%,3\text{mm}}<1$		
*Patient #*	*Prostate*	*Lung*	*Head and Neck*
1	100	99.9	99.4
2	99.9	98.8	99.3
3	100	99.8	99.3
4	100	99.5	99.9
5	100	100	93.2
6	100	100	99.2
7	100	99.8	99.1
8	100	100	99.1
9	100	100	99.1
10	100	99.0	99.0
Average	100±0.0	99.7±0.1	98.7±0.6

## IV. DISCUSSION

The findings of this work are noteworthy in the potential for improving safety and quality in radiation oncology treatment planning, a process that occurs every day in nearly every radiation oncology clinic in the world. In addition to automatically evaluating the dosimetric accuracy and quality of treatment plans, the use of a dose calculation model precommissioned with a reference, or stock, dataset also offers unique and significant advantages. To a first approximation, fundamental beam data characteristics (percentage depths doses and off‐axis ratios) are similar for a given linear accelerator manufacturer and model. It is, therefore, reasonable to assume that a single commissioning dataset would be adequate for verification of all treatment plans from a given linear accelerator model (e.g., Varian iX, Elekta Synergy). In addition to being easier to adopt into clinical practice, a unified reference model also serves to independently verify the TPS beam model and the integrity of its commissioning data. The significance of the latter observation is potentially very high. A recent study by Nelms et al.^(14)^ described eight cases where traditional patient‐specific QA measurements had failed to detect a clinically meaningful dosimetric error; of the eight cases, all resulted from errors in the TPS model, algorithm, or configuration, as opposed to data transfer or deliverability issues. Thus, there is evidence that enhanced tools for treatment plan verification could have a meaningful impact on patient safety in radiation oncology.

The purpose of treatment plan verification or “secondary checks” is to catch errors or mistakes in the treatment plan that could result in harm to the patient. Of potential concern is that the collapsed cone convolution/superposition (CCCS) dose algorithm used by M3D is similar to that used by other TPSs, leading to the hypothesis that such an approach would not be capable of detecting inherent flaws in the TPS algorithm. However, the dosimetric accuracy of convolution‐based algorithms for treatment planning purposes is well documented by a large body of literature under a variety of conditions, compared with measurements and Monte Carlo simulations.^15^, ^16^, ^17^, ^18^, ^19^ Hence, any errors in the dose calculation are likely to result from 1) the specific implementation of the algorithm within a particular system, the permutations of which are large, or 2) a software malfunction (i.e, a “bug”) resulting from an architectural deficiency under specific parameters. As noted by AAPM Task Group 53,^(20)^ a modern planning system may be the result of 30–50 person‐years of work, consisting of 1 million lines of code or more. Even well‐designed and implemented software systems will usually contain at least one software error in every 100–1000 lines of code,^(21)^ some of which will produce significant errors under certain conditions. In such cases, the role of the secondary check is to identify circumstances where the TPS implementation has produced an errant result. This can be achieved either by using a different class of algorithm or by using a different implementation, with the probability of two independent implementations of an algorithm producing identical errors being exceedingly small. In this case, the CCCS algorithm within M3D was developed in‐house and, therefore, uses different approaches to each step of the algorithm (e.g., beam model parameterization, fluence transport, ray tracing, TERMA calculation, output factor determination), meaning it is unlikely that a calculation error in a TPS using a CCCS algorithm would be replicated by M3D.

This work also had several limitations. Both the TPS and M3D dose distributions were extracted from the M3D software. The TPS dose was separately verified to match that taken directly from the clinical server; however, M3D does not contain planar dose export tools that easily facilitate registration of a single dose plane with a measured film image. Thus, while the TPS and M3D dose matrices were always coregistered, aligning them with the film image required data manipulation with custom MATLAB (MathWorks) scripts. The point‐dose agreement was also found to be sensitive to the exact dimensions and placement of the ROI corresponding to the active chamber volume in the CT dataset, particularly for lower dose points located in high‐dose gradient regions. However, as the TPS and M3D and dose distributions showed similar features, a slight change in film registration or ROI shape/location produced a change in agreement between each calculation and measurement that was similar in direction and magnitude. Thus, while agreement of each system with measurement could potentially be improved slightly, the difference in the agreement between the two dose distributions and the measurement would not be expected to change. Finally, it is important to note that only a single TPS and linear accelerator model was examined in this work. Future work should focus on validation of M3D for other clinical vendors. The ability of the system to detect known problems in treatment plans, such as those described in the work of Nelms et al.,^(14)^ should also be assessed.

## V. CONCLUSIONS

The M3D system showed dosimetric accuracy comparable with the TPS and identified several plans that exceeded dosimetric benchmarks. The M3D system possesses the potential to enhance the current treatment plan verification paradigm and improve safety in the clinical treatment planning and review process.

## ACKNOWLEDGMENTS

The author would like to thank Nathan Childress for technical insights regarding the function the Mobius3D treatment plan verification system. Portions of this study were supported by research grants from Mobius Medical Systems, LP, and Elekta, Ltd.
